# Myotonometry and extended field-of-view ultrasound imaging allow reliable quantification of patellar tendon stiffness and length at rest and during maximal load, whereas several restrictions exist for the Achilles tendon

**DOI:** 10.3389/fspor.2024.1379506

**Published:** 2024-05-27

**Authors:** Florian Wegener, Arne Ritterbusch, Christian Saal, Christian Baumgart, Matthias W. Hoppe

**Affiliations:** ^1^ Movement and Training Science, Faculty of Sport Science, Leipzig University, Leipzig, Germany; ^2^ Department of Movement and Training Science, Faculty of Humanities and Social Sciences, University of Wuppertal, Wuppertal, Germany; ^3^ Department of Exercise Science, Institute of Sport Science and Motology, Faculty of Educational Sciences, Philipps University of Marburg, Marburg, Germany

**Keywords:** MyotonPRO, digital palpation device, panoramic, technology, strain, properties, *in vivo*, novice

## Abstract

**Introduction:**

Stiffness and length are well-established tendon parameters in sports and medicine. Myotonometry and ultrasound imaging are the commonly used methods to quantify these parameters. However, further studies are needed to clarify the reliability of these methods, especially when assessing maximally loaded tendons and when conducted by different experienced investigators. This study aimed to determine the intra- and interrater reliabilities of measuring the stiffness and length of the patellar tendon (PT) and Achilles tendon (AT) using the myotonometry method and the extended field-of-view ultrasound (EFOV-US) technique at rest and maximal load performed by different experienced investigators.

**Methods:**

Twenty-seven participants were examined on three different days by one experienced investigator and one novice investigator. Primary outcomes were the intraclass correlation coefficient (ICC) and associated 95% confidence interval (95% CI), coefficient of variation (CV), standard error of measurement (SEM), and minimal detectable change (MDC) across the measurement days and investigators.

**Results:**

For PT measurements at rest and maximal load, the estimated ICCs for stiffness and length were ≥.867 and ≥.970, respectively, with 95% CIs ranging from poor (.306) to excellent (.973) and good (.897) to excellent (.999). The CV, SEM, and MDC for PT stiffness and length were ≤5.2% and ≤2.0%, ≤39.3 N/m and ≤0.9 mm, and ≤108.9 N/m and ≤2.6 mm, respectively. For AT measurements, some restrictions were evident for stiffness at rest and both parameters at maximal load. However, regarding AT length at rest, the estimated ICC was ≥.996, with an excellent 95% CI (.987–.999). The CV, SEM, and MDC for AT length at rest were 2.8%, ≤1.1 mm, and ≤2.9 mm, respectively.

**Conclusion:**

The estimated ICCs show good to excellent reliability for the myotonometry method and the EFOV-US technique for measuring PT stiffness and length at rest and maximal load for experienced and novice investigators. However, some restrictions are evident for the AT, especially for measurements at maximal load.

## 1 Introduction

Tendons are key elements of the musculoskeletal system for generating movement. Their biomechanical and morphological properties allow them to transmit and resist forces (1) and to store and release energy (2). In recent decades, these properties have been investigated in sports and medicine, especially for the patellar (PT) and Achilles tendons (AT) (3, 4). Two commonly quantified parameters are tendon stiffness (i.e., resistance to deformation against external forces) and length (i.e., distance between tendon origin and insertion) (5). For their quantification, force diagnostic and ultrasound (US) imaging methods have been established (6). However, inconsistent application can lead to variations in the mentioned parameters values (6). Another problem in quantifying tendon stiffness is related to the high methodological and mathematical effort involved given the need to know the tendon elongation, forces, and moment arm (7). Furthermore, measuring tendon length using US imaging can be challenging because the tendon origin and insertion should be detected with one field of view. This is particularly problematic in adults as their PTs and ATs are longer than those of commonly used small-sided US probes (6). Thus, more practicable approaches to quantify tendon stiffness and length are needed.

Previous studies have evaluated different practicable approaches for determining tendon stiffness and length. Myotonometry-based approaches have been established concerning stiffness (8). Myotonometry is an indirect and non-invasive method that is based on the analysis of the deformation and oscillations of soft biological tissues induced by a short mechanical impulse applied perpendicular to the tissue surface (8). A frequently used instrument for objective measurements is the MyotonPRO digital palpation device (9, 10), which has the highest estimated intraclass correlation coefficient (ICC), ranging from .94 to .98, compared to other handheld devices for measuring the stiffness of different layers in a phantom tissue model (11). The 95% confidence interval (95% CI) of the estimated ICC indicates a moderate to excellent reliability (.61–1.00) depending on the layer of the phantom tissue (11). For the PT and AT, MyotonPRO stiffness measurements show an estimated ICC ranging from .51 to .98 depending on the type of comparison (e.g., intra- or interrater reliability), knee and foot joint angles, and load state of the tendon during the measurements (12–16). Regarding the 95% CI of the estimated ICC, the reliability varied from poor to excellent (.29–.99) depending on the measurement conditions mentioned. Unfortunately, not all studies reported a 95% CI. For the length measurement of larger musculoskeletal structures, US imaging using the extended field-of-view (EFOV) technique has recently been suggested as a practicable approach (17). This technique allows the acquisition of US images larger than the field of view considering multiple and later aggregated images as the probe moves over the tissue (17). Previous studies have shown that the EFOV-US technique has excellent intra- and interrater reliabilities for distance measurements in the phantom tissue, with an estimated ICC of .998 (95% CI .992–.999) (18). Although no study has yet evaluated the reliability of the EFOV-US technique for PT length measurements, previous studies have shown that the technique has a moderate to excellent test–retest reliability, with an estimated ICC of .830–.954 (95% CI .728–.983) for measurements at the AT (19–21). In general, the myotonometry method and the EFOV-US technique can be considered as practical and reliable approaches for estimating tendon stiffness and length, respectively, but the measurement conditions (e.g., tendon loading state and body position during the measurements) and the type of comparison (e.g., intra- or interrater) influence the reliability indices of the measurements.

However, previous reliability studies of PT and AT stiffness measurements using myotonometry with MyotonPRO have only been performed on resting or submaximal loaded (≤70% of the maximal isometric force) tendons (12–16). Similarly, reliability studies AT length using the EFOV-US technique have only been performed at rest (19–21), and there are no studies available for PT yet. Mechanical loading influences the reliability of stiffness measurements using myotonometry (15) and causes tendon elongations (22); hence, it is worthwhile to evaluate the reliability of the myotonometry method and the EFOV-US technique for maximal loaded PTs and ATs. Additionally, US imaging in the aforementioned studies was performed by experienced investigators only. Due to the fast learning curve in the field of US imaging (23, 24), it is also rational to question the reliability of the EFOV-US technique when used by novices. Overall, previous reliability studies on PT or AT stiffness and length using the myotonometry method and the EFOV-US technique, respectively, lack some aspects that are important for use in sports and medicine. Therefore, this study aimed to evaluate, for the first time, the intra- and interrater reliabilities of PT and AT stiffness and length measured using the myotonometry method and the EFOV-US technique at rest and maximal load performed by different experienced investigators.

## 2 Methods

### 2.1 Participants and ethics statement

The required sample size was estimated by a web-based calculator for reliability studies according to Arifin (25). Therefore, the following parameters were used: *p*0 = .75, *p*1 = .95, *α *= .05, and power = 80.0%. Considering a dropout rate of 5%, the calculated sample size was *n* = 13. The study was conducted between June 2022 and February 2023. The participants were students and staff of the Faculty of Sports Science at Leipzig University, who were recruited through personal contacts. For study inclusion, the participants had to be of legal age (≥18 years), sportively active (≥1 training session per week), and free of acute musculoskeletal injuries or infections. The participants were informed of the study aim, potential risks, applied procedures, and the right to withdraw from the study at any time. Subsequently, the participants provided written informed consent to participate in the study prior to the examinations. A total of 27 participants were recruited and assigned to PT (*n* = 14; female = 8; age, 23.1 ± 3.0 years; body height, 1.76 ± 0.08 m; body mass, 70.6 ± 8.9 kg) and AT (*n* = 13; female = 7; age, 27.8 ± 5.3 years; body height, 1.74 ± 0.05 m; body mass, 69.8 ± 5.4 kg) groups in the order of recruitment until the required sample size was reached. All procedures were preapproved by the Ethics Committee of Leipzig University (2022.02.23_eb_137) without any revisions. All methods were performed in accordance with the relevant guidelines and regulations.

### 2.2 Experimental design and study protocol

**Figure 1:**
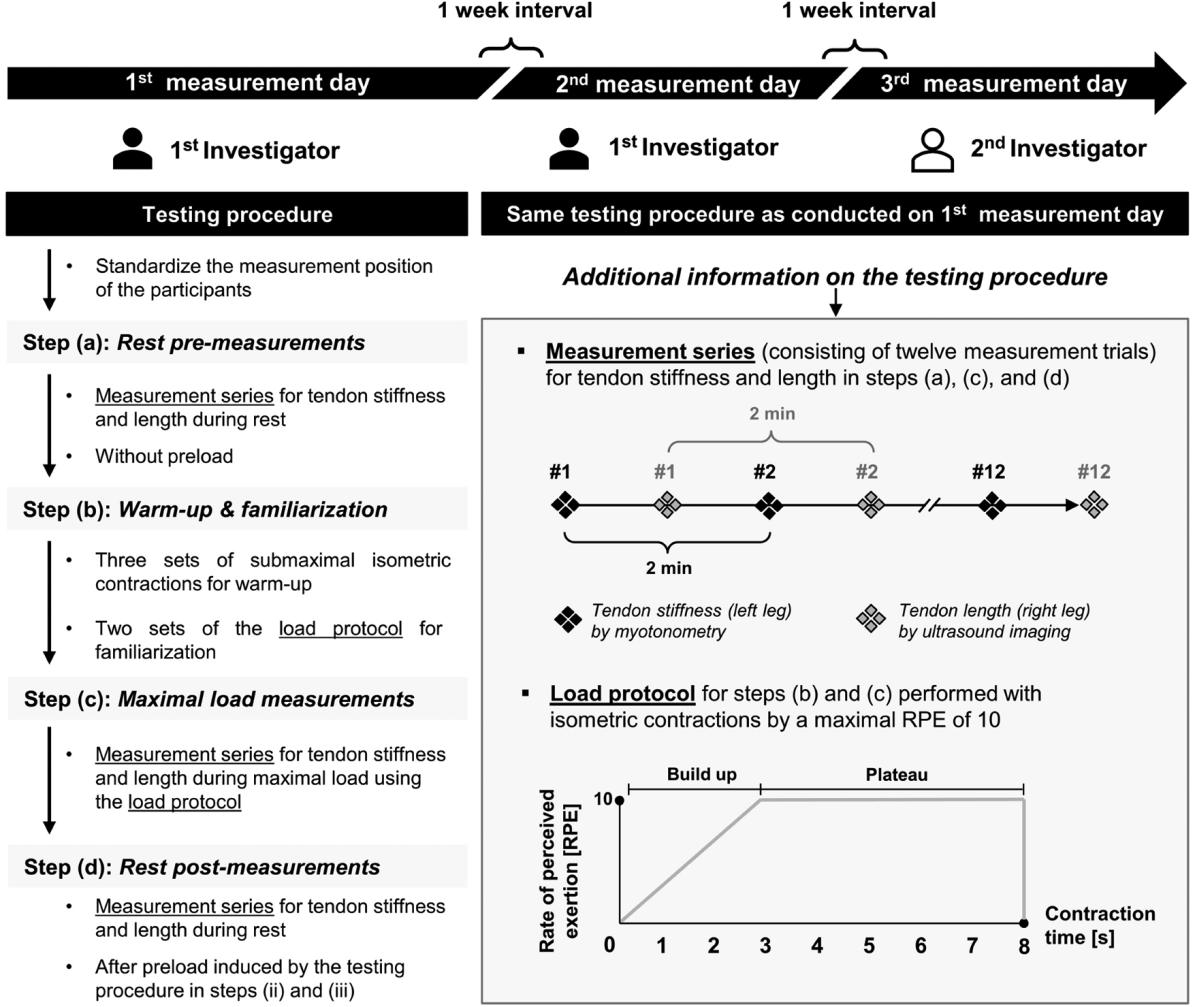
Research design and testing procedure. Patellar and Achilles tendon groups were independently investigated using the same standardized testing procedures. Both investigators were certified physiotherapists and sports scientists with different experiences in ultrasound imaging. The first investigator (colored in black) is experienced, and the second investigator (colored in white) is a novice, who was instructed prior to the study. Min, minutes; s, seconds.

To evaluate the intra- and interrater reliabilities of PT and AT stiffness and length measured using the myotonometry method and the EFOV-US technique at rest and maximal load performed by different experienced investigators, respectively, all participants in each group were examined on three different days separated by 1 week. The test-retest reliability was estimated between these sessions. The measurements of the individual participants were conducted at the same time of day, and both groups were independently investigated using the same standardized testing procedure. One investigator performed the measurements on the first 2 days (first investigator) and the other performed the measurements independently on the third day (second investigator). Both investigators are certified physical therapists and sports scientists. Prior to the study, the first investigator attended a sonography course (DEGUM course ID: 9079) for sports traumatology, had more than 1 year of experience with US imaging of PTs and ATs, and instructed the second investigator, who was a novice in US imaging and the use of MyotonPRO. The primary outcomes were the estimated ICC and associated 95% CI for the intra- and interrater reliabilities, overall coefficient of variation (CV), standard error of measurement (SEM), and minimal detectable changes (MDC) for PT and AT stiffness and length measurements obtained during rest and maximal load. More information on the primary outcomes is provided in the statistical analysis section. As secondary outcomes, the ICC for the calculated tendon elongation was considered and the possible influence of the applied load on PT and AT stiffness and length resting values induced by the test procedure was proven. Figure 1 shows the research design and the testing procedure.

For the testing procedure, as previously described, the measurements were taken on a treatment table in a seated position with a hip and knee joint flexion angle of 90° for the PT group (12, 26) and in a prone position with a neutral ankle position (90°) for the AT group (15, 19). In addition, straps were placed anteriorly on the proximal and distal thighs for the PT group and posteriorly on the distal thighs for the AT group to fix the participant and restrict degrees of freedom. To avoid movements of the whole body during the maximal tendon load in the AT group, further fixation straps were placed over the trunk and shoulder and attached to the treatment table. To perform isometric maximal voluntary contractions, the cuffs were secured to the treatment table with non-elastic straps parallel to the floor and placed around the ankles for the PT group and over the forefeet for the AT group. The testing procedure was identical for each of the three measurement days and consisted of four steps: rest pre-measurements (a), warm-up and familiarization (b), maximal load measurements by isometric maximal voluntary contractions (c), and rest post-measurements (d). In steps a, c, and d, stiffness and length were measured as measurement series alternately for the left and right legs. A 2-min passive recovery period was set between the measurements on the same leg, during which the other parameter was measured on the contralateral leg. For high reliability in measuring the tendon properties, averaging five to six measurements per parameter is recommended (27). PT and AT stiffness and length were measured for the first time in our study during the maximal load using myotonometry and the EFOV-US technique, respectively, and pilot tests showed a different number of trials to record five measurements for the PT; hence, each measurement series was set to a high number of 12 measurement trials per parameter. Additionally, the pilot tests showed some difficulties in measuring both parameters during the maximal load in the AT group. To standardize the testing procedure, the same protocol was used for measuring stiffness and length at rest and maximal load in both the PT and AT groups. However, each measurement day started with the rest pre-measurements (a), which were followed by warm-up and familiarization (b). As previously described, the warm-up consisted of three sets of submaximal isometric knee extension or plantar flexion contractions for the PT or AT groups (27, 28), respectively, alternating between the left and right legs. The three warm-up sets consisted of 1 s of isometric contraction per leg with 10, seven, and five repetitions, separately, and 1 min of passive recovery between the sets. The intensity was predefined by a modified rate of perceived exertion (RPE) scale ranging from 0 (minimal) to 10 (maximal) and set at 3–4, 5–6, and 7–8 for the three sets, separately. At the end of the warm-up, as recommended for familiarization and to ensure the necessary conditioning of the tendons (29), the load protocol used for the measurements at the maximal load was introduced and performed alternately two times per leg, with 2 min of passive recovery between the same leg. The load protocol for the maximal load measurements is shown in Figure 1. The load protocol was performed with isometric contractions and consisted of two phases. First, a progressive increase in load from RPE 0 to 10 was performed in the first 3 s; then, the maximal RPE of 10 was maintained at a plateau for 5 s. After warm-up and familiarization, the maximal load measurements were performed with isometric maximal voluntary contractions (c), which were conducted during the 5 s of the plateau phase of the load protocol. Finally, rest post-measurements (d) were performed to analyze the influence of the applied load induced by steps (b) and (c) on the parameter resting values. A video with audio signals was programmed for all experimental steps to standardize and guide the participants and investigators throughout the data collection.

### 2.3 Equipment

The measurement of tendon stiffness using myotonometry was conducted with a MyotonPRO digital palpation device (Myton AS, Tallinn, Estonia). The measurement points were 2 cm below the apex patella and on a connecting line between the tip of the medial and lateral malleoli in the center of the PT and AT, respectively. The points were marked with a pen by the respective investigator on each of the three measurement days where the measuring points from the previous sessions were no longer visible. For application, the MyotonPRO probe was held perpendicular to these points with a predefined pressure of 0.18 N, as shown by a light signal on the probe. Short mechanical impulses of 15 ms and 0.42 N were released by the device, resulting in deformation and rebound of the underlying tissue. This rebound was measured using internal accelerometers. The stiffness (N/m) was calculated $\left(a_{\max}\times m_{\mathrm{probe}}/\Delta l\right)$ based on the acceleration (a_max_), predefined pressure (m_probe_), and difference in the probe length at rebound (Δl) (30). As shown to be valid, compared to the standard protocol of lower limb stiffness measurements (31), one measurement was conducted with five successive pulses at an interval of 0.8 s, after which the device displayed the average stiffness with the corresponding CV. The manufacturer recommends repeating the measurements when the CV is >3%. The overall CV was calculated manually as one of the primary outcomes; therefore, the displayed CV of the device was not considered.

**Figure 2:**
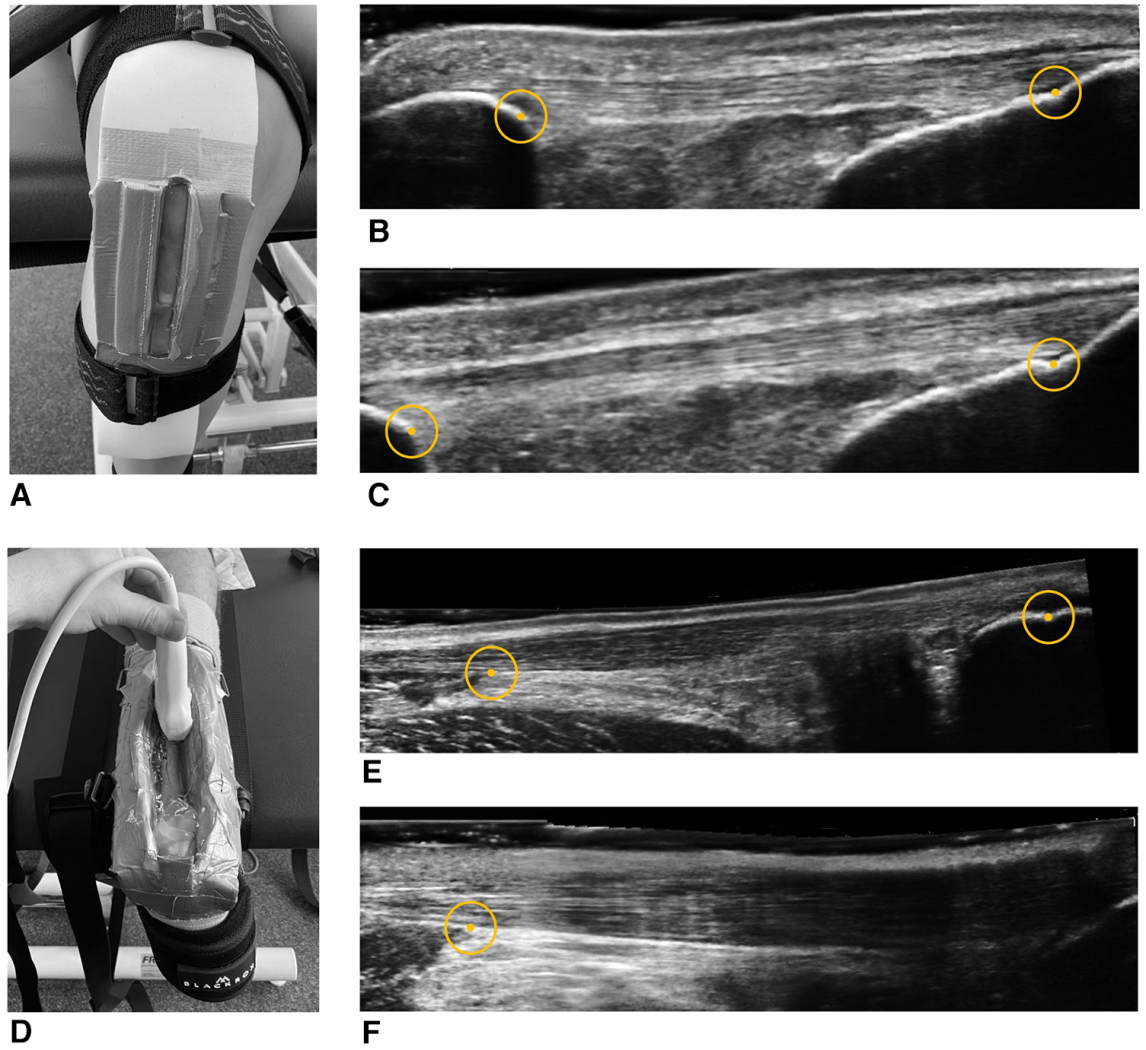
Self-customized fixable brace and anatomical reference points for ultrasound imaging and length measurement of the patellar and Achilles tendons. Ultrasound images of the patellar (**A**) and Achilles tendons (**D**) at rest (**B**,**E**) and maximal load (**C**,**F**), respectively. For point-to-point length measurements, the circles show the anatomical reference points (apex patella, tuberosity tibiae, M. soleus, and calcaneus notch). (**F**) Example of the common unclear reference point of the calcaneus at the maximal load measurements.

The measurement of tendon length using the EFOV-US technique was conducted with the portable B-mode US imaging device, SonoScape E2 (SonoScape Medical Corporation, Shenzhen, China). As described previously, the anatomical reference points were the last visible point of the apex patella, the deep tuberosity tibiae, the musculotendinous junction of the M. soleus, and the calcaneus notch for the PT and AT, respectively (20, 27). For imaging, an L741 linear array (frequency, 4.0–16.0 MHz; field of view, 46 mm) with a preprogrammed configuration from the manufacturer for the imaging of musculoskeletal structures (depth, 4.5 cm; frequency, 9.5–12.2 MHz; power, 100.0%) was used. The length measurements were conducted point-by-point between the anatomical reference points using integrated caliper software. For practical application, after starting the EFOV-US mode, the probe was pulled slowly (1 cm/s) longitudinally along the tendon axis. Excessively fast and slow movements of the probe were displayed on the screen of the US device. To prevent axial deviations of the probe, we used a self-customized fixable brace for standardization. Figure 2 shows the self-customized fixable brace and the anatomical reference points of US imaging for the length measurement of the PT and AT.

### 2.4 Data processing and statistical analysis

As previously recommended, for further analysis, tendon stiffness and length at rest and maximal load were calculated from the mean value of five measurement trials per parameter (27). For tendon stiffness, the measurement trials, in which the MyotonPRO showed an error during the measurement, could not be used because no values were displayed. For tendon length, the measurement trials were not considered if the US images showed unclear anatomical reference points or if the screen of the US device was frozen at any time during the EFOV-US imaging process. The first five measurement trials of the respective measurement series that were not rejected based on these criteria were used for the calculation of the mean and further statistical analysis. If fewer than five measurement trials were available per measurement series, this measurement series for the respective parameter was discarded for further statistical analysis. The results of the first investigator's measurements between measurement days 1 and 2 were processed independently of each other. The investigator was blinded to the second investigator until statistical analysis, which was performed by the first investigator.

All statistical calculations were performed using SPSS software version 28 (IBM SPSS Statistics for Windows, Armonk, NY: IBM Corp). Reliability was tested for the calculated means of the five measurement trials of PT and AT stiffness and length between two measurement time points using measurements obtained on days 1 and 2 for the intrarater reliability and measurements obtained on days 2 and 3 for the interrater reliability. The measurement time points for the interrater reliability were chosen because the interval between these measurements was shorter than that between measurement days 1 and 3. Therefore, the estimated ICCs and 95% CIs were calculated based on the mean rating (*k* = 5) and the absolute agreement with two-way mixed-effects and two-way random-effects models for the intrarater (same investigator, ICC 3,5) and interrater reliabilities (two different investigators, ICC 2,2) of the parameters. According to Koo and Li (32), reliability was considered poor, moderate, good, or excellent when the 95% CIs of the estimated ICCs were <.50, .50–.75, .75–.90, and >.90, respectively. Additionally, a paired *t*-test with effect sizes according to Cohen's *d* was conducted to evaluate whether there were any statistically significant differences in PT and AT stiffness or length between measurement days 1 and 2 or between measurement days 2 and 3 due to the given normal distribution of all parameters, as shown by the Kolmogorov‒Smirnov test (*p* > .05). The overall CV for PT and AT stiffness and length at rest and maximal load measurements was calculated as the average of the individual CV over all participants. For this, the CV for the three measurement days was estimated for each of the five included measurement trials of the respective parameter by dividing the standard deviation (SD) by the mean value. The SEM and MDC were calculated as previously described using SD×√(1−ICC)and1.96×√2×SEM, respectively (12). For the SD calculation, the mean values of the first two and last two measurement days of each participant and the estimated ICCs for the intra- and interrater reliabilities were considered. For tendon elongation and strain, the difference in the length of the maximal load measurements (c) and rest pre-measurements (a) was calculated and related to the resting length. To test for a possible influence of the applied load induced by steps (b) and (c) on tendon stiffness and length resting values, a comparison between rest pre-measurements (a) and rest post-measurements (d) was conducted. For this analysis, a paired *t*-test was used. The significance level for all the statistical tests was set at *p* < .05.

## 3 Results

No participant withdrew from the study. However, in the PT group (*n* = 14), one participant was not available on the third measurement day due to illness. Thus, of the 42 planned measurement days, 41 (97.7%) were carried out. Additionally, the measurement series for the tendon length at rest could not be collected for one participant on the first measurement day due to technical problems, but all other parameters were considered. In the AT group (*n* = 13), all participants attended the scheduled 39 measurement days (100.0%). Table 1 summarizes the descriptive data for PT and AT stiffness and length during rest pre- and maximal load measurements.

**Table 1:** Descriptive data for patellar and Achilles tendon stiffness and length during rest pre- and maximal load measurements.

Patellar tendon	Measurement day 1 (Investigator 1) Mean ± SD	Measurement day 2 (Investigator 1) Mean ± SD	Measurement day 3 (Investigator 2) Mean ± SD	Difference from days 1–2 Mean ± SD	Difference from days 2–3 Mean ± SD
Stiffness rest pre-measurements (N/m)	794.3 ± 101.4 (n = 14)	790.6 ± 99.4 (n = 14)	780.7 ± 98.2 (n = 13)	4.7% ± 5.0% (n = 14)	4.8% ± 5.4% (n = 13)
Stiffness maximal load measurements (N/m)	929.4 ± 122.5 (n = 12)	881.4 ± 85.8 (n = 10)	857.0 ± 65.4 (n = 8)	6.2% ± 4.7% (n = 10)	3.6% ± 3.7% (n = 8)
Length rest pre-measurements (mm)	46.1 ± 5.2 (n = 13)	46.2 ± 4.9 (n = 14)	46.0 ± 4.8 (n = 13)	1.2% ± 0.7% (n = 13)	2.2% ± 1.1% (n = 13)
Length maximal load measurements (mm)	49.5 ± 5.2 (n = 14)	49.5 ± 4.8 (n = 14)	50.1 ± 5.9 (n = 13)	1.5% ± 1.2% (n = 14)	2.7% ± 2.4% (n = 13)
Achilles tendon					
Stiffness rest pre-measurements (N/m)	1,114.2 ± 43.9 (n = 13)	1,098.3 ± 39.6 (n = 13)	1,056.9 ± 60.2 (n = 13)	3.2% ± 1.4% (n = 13)	5.6% ± 4.3% (n = 13)
Stiffness maximal load measurements (N/m)	Insufficient data (n ≤ 6 per measurement day, but different participants per day)				
Length rest pre-measurements (mm)	57.6 ± 16.3 (n = 13)	57.6 ± 17.3 (n = 13)	56.9 ± 17.0 (n = 13)	2.7% ± 2.5% (n = 13)	2.6% ± 2.1% (n = 13)
Length maximal load measurements (mm)	Insufficient data (n = 1 per measurement day, but different participants per day)				

The absolute values shown are calculated from the mean values of the measurement series per participant for each parameter and measurement condition. The differences between measurement days are reported as absolute relative differences. This represents the average of the percentage differences of the respective measurement days of each participant regardless of the direction. n, number of participants whose measurement series could be included per measurement day; *n*, number of available data pairs; N/m, Newton per meter; mm, millimeter; and SD, standard deviation.

### 3.1 Patellar tendon

**Figure 3:**
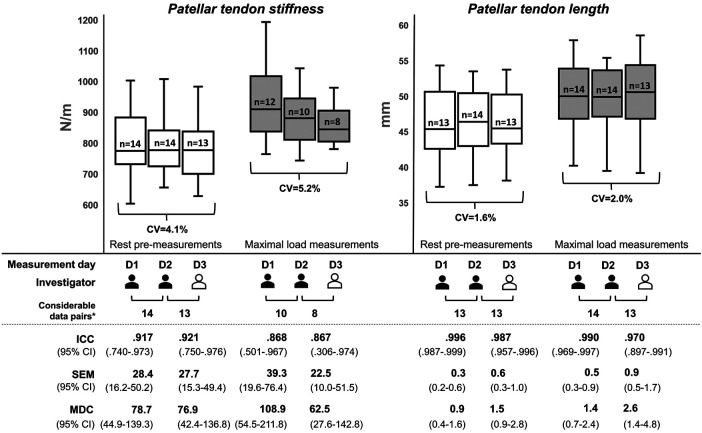
Reliability indices for patellar tendon stiffness and length during rest pre- and maximal load measurements. The box plots were built using the mean from five measurement trials for each parameter and measurement series per participant. The investigators differed in their experience with ultrasound imaging, with the first investigator (colored in black) being experienced and the second investigator (colored in white) being a novice, who was instructed prior to the study. D, measurement day; CV, coefficient of variation; ICC, intraclass correlation coefficient; MDC, minimal detectable change; SEM, standard error of the measurement; n, number of participants with valid measurement series; *number of data pairs that could be recorded from the same participant on both respective measurement days.

Figure 3 shows the box plots and reliability indices for PT stiffness and length during rest pre- (a) and maximal load measurements (c). There were no significant differences (*p* ≥ *.*097; *d* ≤ *.*499) between the same parameters and conditions for days 1 and 2 or days 2 and 3. For the calculation of the ICCs of the intrarater reliability for the rest and maximal load measurements, the numbers of considerable data pairs were 14 and 10 for PT stiffness and 13 and 14 for PT length. For the interrater reliability, the numbers were 13 and 8 and 13 and 13, respectively. As primary outcomes, the intra- and interrater reliabilities for the resting measurements of PT stiffness were moderate to excellent with an estimated ICC ≥.917. For the maximal load measurements, the intra- and interrater reliabilities were moderate to excellent and poor to excellent, respectively, with an estimated ICC ≥.867. The overall CV, SEM (95% CI), and MDC (95% CI) for both the investigators and measurement conditions were ≤5.2%, ≤39.3 (10.0–76.4) N/m, and ≤108.9 (27.6–211.8) N/m, respectively. For PT length at rest and maximal load measurements, the intra- and interrater reliabilities were good to excellent, with an estimated ICC ≥.970 and a lower limit of the 95% CI falling below the threshold for excellent reliability only for the interrater comparison during the maximal load measurements. The overall CV, SEM (95% CI), and MDC (95% CI) for both the investigators and measurement conditions were ≤2.0%, ≤0.9 (0.2–1.7) mm, and ≤2.6 (0.4–4.8) mm, respectively.

As secondary outcomes, the average calculated PT elongation and strain were 3.4 ± 0.6 mm and 7.4% ± 1.3%, 3.3 ± 0.8 mm and 7.3% ± 2.1%, and 4.1 ± 1.6 mm and 8.7% ± 3.2% for days 1, 2, and 3, respectively. The intra- and interrater reliabilities regarding the calculated tendon strain were poor to excellent and poor to good, with estimated ICCs of .734 and .369, respectively. The paired *t*-tests between the rest pre-measurements (a) and post-measurements (d) for each of the three measurement days showed no significant differences in PT stiffness (*p* ≥ .107; *d* ≤ *.*483) or length (*p* ≥ *.*424; *d* ≤ *.*229).

### 3.2 Achilles tendon

**Figure 4:**
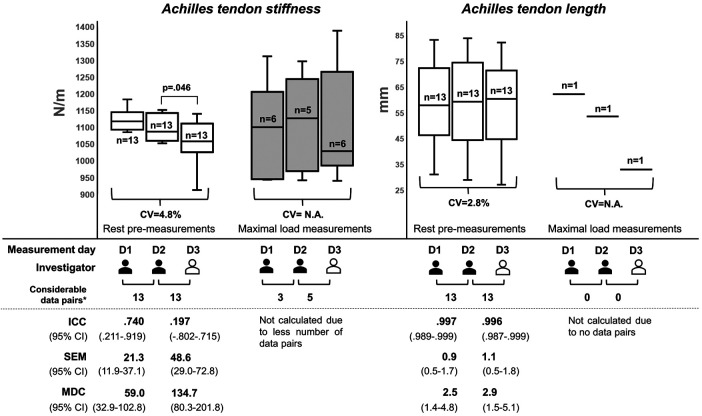
Reliability indices for Achilles tendon stiffness and length during rest pre- and maximal load measurements. The box plots were built using the mean from the five measurement trials for each parameter and measurement series per participant. The investigators have different experiences in ultrasound imaging. The first investigator (colored in black) is experienced, while the second investigator (colored in white) is a novice, who was instructed prior to the study. D, measurement day; CV, coefficient of variation; ICC, intraclass correlation coefficient; MDC, minimal detectable change; N.A., not enough data for statistical analysis available; SEM, standard error of measurement; n, number of participants with valid measurement series*number of data pairs that could be recorded from the same participant on both of the respective measurement days.

Figure 4 shows the box plots and reliability indices for AT stiffness and length during the rest pre-measurements (a). The visualized data for the maximal load measurements (c) were not meaningful due to the small number of considerable data pairs. Therefore, no maximal load measurements were considered for further statistical analysis of AT stiffness and length. For the rest pre-measurements, there was a significant difference (*p* = .046; *d* = *.*619) in AT stiffness between days 2 and 3. No further significant differences (*p* ≥ *.*148; *d* ≤ *.*429) were found between the same parameters and conditions for days 1 and 2 or days 2 and 3. For the calculation of the ICCs for the intra- and interrater reliabilities, there were 13 considerable data pairs for both AT stiffness and length. As primary outcomes, the intra- and interrater reliabilities for resting measurements of AT stiffness were poor to excellent and poor to moderate, with estimated ICCs of .740 and .197, respectively. The overall CV, SEM (95% CI), and MDC (95% CI) for both investigators were 4.8%, ≤48.6 (11.9–72.8) N/m, and ≤134.7 (32.9–201.8) N/m, respectively. For AT length measurements at rest, the intra- and interrater reliabilities were excellent, with an estimated ICC ≥.996. The overall CV, SEM (95% CI), and MDC (95% CI) for both the investigators were 2.8%, ≤1.1 (0.5–1.8) mm, and ≤2.9 (1.4–5.1) mm, respectively.

The AT strain could not be calculated due to the large number of invalid measurement series of the tendon length at the maximal load. The paired *t*-tests between the rest pre-measurements (a) and post-measurements (d) on each of the three measurement days showed no significant differences in AT stiffness (*p* ≥ *.*218; *d* ≤ *.*360) but demonstrated a tendency in AT length (*p* ≥ *.*05; *d* ≤ *.*603).

## 4 Discussion

This study aimed to evaluate for the first time the intra- and interrater reliabilities of PT and AT stiffness and length measured using the myotonometry method and the EFOV-US technique at rest and maximal load performed by different experienced investigators, respectively. The main findings were that (i) the estimated ICCs were good to excellent for PT measurements, independent of the investigator's experience, whereas (ii) some restrictions are evident for AT measurements.

### 4.1 Main findings

The first main finding was that, independent of the investigator's experience, the estimated ICCs were good to excellent for PT measurements (Figure 3). For the PT stiffness rest measurements, the findings for the intra- and interrater reliabilities are consistent with those of previous studies showing estimated ICC values ≥.80 (12–14). Nevertheless, the 95% CI in this study indicated a range from moderate to excellent reliability, which was also shown in the study by Chen et al. (12). However, there is still a lack of research on PT stiffness measurements using myotonometry for maximally loaded tendons (31). With this in mind, our study showed good estimated ICCs for the intra- and interrater reliabilities, but these results should be interpreted with caution due to the measurement error-related dropouts for a few data pairs, resulting in a smaller than the calculated sample size, and the 95% CIs indicated a large range from poor to excellent reliability. It should also be noted that myotonometry-based measurements record the transversal stiffness at the local measurement site of the tendon and could be affected by numerous tendon-related parameters (33). Therefore, it should be emphasized that stiffness measurements using myotonometry at loading conditions cannot be considered interchangeable with traditional procedures using synchronized force and ultrasound diagnostics (6). However, for measurements of PT length at rest and maximal load, our study demonstrated, for the first time, excellent estimated ICCs for the intra- and interrater reliabilities for experienced and novice investigators using the EFOV-US technique. In addition, the 95% CI indicated excellent reliability, except for the interrater reliability at the maximal load, which demonstrated good (but bordering excellent) to excellent reliability. This finding speaks to a fast learning curve for the EFOV-US technique, as has been shown previously for other US-based approaches (23, 24) and provides a practical framework to investigate PT length in different contexts, such as growth changes (34), pathological tendon conditions (3), and effect of mechanical loading (35). Additionally, the calculated tendon strain showed good ICC values for intrarater reliability, but the 95% CIs ranged from poor to excellent. It should be noted that this parameter was calculated from the measurements of two static ultrasound images rather than from a single dynamic ultrasound video, as is usually the case (7); however, the calculated values were comparable to those of previous studies (36–38). Taken together, these findings suggest that, independent of the investigator's experience, the myotonometry method using MyotonPRO and the EFOV-US technique are reliable and practicable for the measurement of PT stiffness at rest and PT length at rest and maximal load, respectively. However, compared to PT stiffness measurements at rest, the maximal isometric contraction-induced loads extended the 95% CI interval and decreased the reliability of the myotonometry method. Further studies are needed to investigate whether or not other standardization measures would lead to more reliable results for PT stiffness maximal load measurements.

Concerning the second main finding, some restrictions are evident for AT measurements with regard to stiffness at rest and for both parameters at maximal load (Figure 4). For AT stiffness measurements at rest, the estimated ICC showed moderate (but bordering good, .740) intrarater reliability, but the 95% CI indicated poor to excellent variability. For the interrater reliability, the estimated ICC was poor (.197), ranging from poor to moderate. This is partly contrary to that in previous studies. Regarding the same measurement position and joint angle, the study by Schneebeli et al. (15) showed an estimated ICC ranging from .76 to .95. The 95% CIs indicated excellent, moderate to good, and good to excellent intrarater, interrater, and intersession reliabilities, respectively. Another study by Pruyn et al. (16) showed an estimated ICC of .72, and the 95% CI indicated poor to good reliability for test–retest comparisons, which is consistent with our results. However, note that the measurements by Schneebeli et al. (15) were conducted with a stable foot plate for ankle fixation. In this work, we used manually adjustable straps for foot standardization in a neutral position, which may result in minimally different joint angles. The different applied pre-tensions on the tendon across the three sessions could have influenced AT stiffness (39). AT stiffness was shown to be more reliable; hence, the measurement of AT stiffness in a relaxed foot position should be considered in further studies (13, 15). However, in line with the PT, there is still a lack of research on AT stiffness measurements at maximal loads. In this context, only a small number of measurement series had been acquired in our study, and no statistical analysis was conducted due to fewer available data pairs between the measurement days. Previous studies have shown a wide range of estimated ICCs (.54–.94) for AT stiffness measured using MyotonPRO during submaximal loads of different intensities (≤70% of the maximal isometric contraction), where the 95% CIs showed poor to excellent reliability (15, 16). This indicates that the magnitude of the load influences AT stiffness as measured through myotonometry. Thus, this method is not reliable for AT stiffness measurements at maximal loads. Our results regarding AT length at rest are consistent with those of previous studies, in which the estimated ICCs were ≥.83, and the 95% CIs showed moderate to excellent reliability (19–21). It has been shown that more reliable measurement results were obtained with a fixable brace. In addition, the US imaging in previous studies was performed by experienced investigators, which, in line with the results of PT length measurements, suggests the fast learning curve of the EFOV-US technique for novices. Again, there is still a lack of research on AT length measurements at maximal loads. There is only one previous study (40) that used the EFOV-US technique to calculate the *in vivo* AT strain, but AT length measurements were only conducted at submaximal loads, and no estimated ICC values were reported. In this study, we were unable to acquire a sufficient number of US images with a sufficient quality of the visualized anatomical reference points. In contrast to the PT, the EFOV-US technique is not reliable for measuring AT length at the maximal load. The potential reason for this was the unavoidable movements of the AT and calcaneus during the maximal load (41, 42), which resulted in a concave deformation of the skin surface and led to a disturbance in the image processing of the EFOV-US technique. Taken together, previous findings suggest that the myotonometry method using MyotonPRO provides reliable measurements of AT stiffness at rest, and our study highlights the need to consider sufficient standardization in terms of the joint ankle position. The EFOV-US technique provides reliable measurements of AT length at rest by experienced and novice investigators when a fixable brace is used.

### 4.2 Clinical and practical implications

Previous studies have shown that PT and AT stiffness and length can be affected by numerous factors, including the maturation process and sex (34, 43), pathological tendon conditions (3, 38, 44), and specific load interventions in the short and long terms (35, 45, 46). This indicates that these parameters may be useful for clinical point-of-care diagnostics in sports or medicine. To evaluate and interpret differences in these parameter values, it is important to know the MDC of the respective measurement method, which is defined as the “minimal change that falls outside the measurement error in the score of an instrument used” (47). Our study (Figures 3, 4) and previous studies reported MDCs for PT and AT stiffness and length measurements using the myotonometry method (12, 13, 15) and the EFOV-US technique (19, 20), respectively. With regard to the results of previous studies investigating changes in PT and AT stiffness (31, 48–53) and length (34, 36–38, 43, 44, 54) in sports and medicine, these changes could be detected outside the measurement error using myotonometry and the EFOV-US technique, which supports the practical applicability of both technologies. For future studies using the EFOV-US technique, and as conducted here, we recommend the usage of a fixable brace to avoid axial shifts of the US probe. This could also be helpful in clinical settings when standardizing the imaging of larger musculoskeletal structures using the EFOV-US technique. Furthermore, sufficient fixation of the joint position should be ensured, especially for ATs. To standardize the measurement procedure in this reliability study, we used the same protocol for both groups at rest and maximal load measurements, which is not necessary for application in sports or medicine. Thus, for the resting PT and AT parameters for which excellent reliability has been shown, a 2-min break between multiple measurements is not needed.

## 5 Limitations and outlook

Our study could be limited by the chosen body and joint fixation methods employed for the measurements during the maximal load due to the use of self-adjusted straps and cuffs. Additionally, we did not determine the torque during maximal isometric contraction. Therefore, we did not quantify the muscular output. Instead, we set the intensity based on the maximal RPE. The reason for our approach was to offer a highly practicable measurement approach, which is important for its real-world application in sports or medicine. However, the results for the measurements at the maximal load should be interpreted with caution. Nevertheless, this limitation does not affect measurements at rest, for which we have shown for the first time that the EFOV-US technique is reliable for measuring PT length. Furthermore, we also showed that reliable results could be generated for PT and AT length by novice investigators. Although this study increases the knowledge of the reliability of PT and AT stiffness and length measurements using the myotonometry method and the EFOV-US technique, respectively, it is important to note that the validity has not been fully clarified yet. Myotonometry using MyotonPRO has been validated for AT stiffness in terms of construct validity (16), while the EFOV-US technique has been validated for AT length in terms of criterion-referenced validity (20), both for measurements at rest only. More studies are needed to verify the validity of the measurements at PT and to consider the measurements at the maximal load. Additionally, further studies are needed to investigate the reliability of both methods for measurements at maximally loaded tendons. It is also worth considering the impact of the learning process of novices and taking the intrarater reliability of both novice and experienced investigators into account. Furthermore, the clinical and practical relevance of myotonometry-based tendon stiffness measurements during mechanical loading in real-world applications has to be investigated.

## 6 Conclusion

The estimated ICCs show good to excellent reliability for the myotonometry method with MyotonPRO and the EFOV-US technique for measuring PT stiffness and length at rest and maximal load for experienced and novice investigators. However, some restrictions are evident for the AT, especially for measurements at the maximal load. More research to clarify the validity and influence of the load magnitude is needed.

## Data Availability

The raw data supporting the conclusions of this article will be made available by the authors, without undue reservation.
